# ProgClust: A progressive clustering method to identify cell populations

**DOI:** 10.3389/fgene.2023.1183099

**Published:** 2023-04-06

**Authors:** Han Li, Ying Wang, Yongxuan Lai, Feng Zeng, Fan Yang

**Affiliations:** ^1^ Department of Automation, Xiamen University, Xiamen, China; ^2^ National Institute for Data Science in Health and Medicine, Xiamen University, Xiamen, China; ^3^ Xiamen Key Lab Big Data Intelligent Anal and Decis, Xiamen, China; ^4^ School of Informatics, Xiamen University, Xiamen, China

**Keywords:** ScRNA-seq, single-cell clustering, ensemble clustering, rare cell, unbalanced data

## Abstract

Identifying different types of cells in scRNA-seq data is a critical task in single-cell data analysis. In this paper, we propose a method called ProgClust for the decomposition of cell populations and detection of rare cells. ProgClust represents the single-cell data with clustering trees where a progressive searching method is designed to select cell population-specific genes and cluster cells. The obtained trees reveal the structure of both abundant cell populations and rare cell populations. Additionally, it can automatically determine the number of clusters. Experimental results show that ProgClust outperforms the baseline method and is capable of accurately identifying both common and rare cells. Moreover, when applied to real unlabeled data, it reveals potential cell subpopulations which provides clues for further exploration. In summary, ProgClust shows potential in identifying subpopulations of complex single-cell data.

## 1 Introduction

Single-cell RNA sequencing (scRNA-seq) emerges as a powerful tool for the exploration of genetic contents in single cells (Picelli et al., 2014; Hedlund and Deng, 2018; Aldridge and Teichmann, 2020). Through sequencing each single cell, biologists can dissect cellular heterogeneity more straightly, and help establish an in-depth understanding of complex cell systems.

Single-cell Clustering that groups cells into cell populations is an essential step toward single-cell analysis (Andrews and Hemberg, 2018; Kiselev et al., 2019; Petegrosso et al., 2020). However, the unbalanced type distributions of scRNA-seq data pose great challenge for clustering-based cell type identification. Major cell types among these can be characterised comprehensively with toolkits such as Seurat and Scater, whereas the rare cell types, making up the majority of the new cell types, remain to be uncovered by specialised approaches. Several single-cell clustering algorithms have been developed (Wang et al., 2017; Hu et al., 2019). For example, SC3 (Kiselev et al., 2017) provides an ensemble approach to combine multiple clustering solutions to yield a consensus clustering. Although SC3 can obtain abundant cell populations accurately and robustly, it tends to fail in identifying non-abundant cell populations, i.e., rare cells.

Rare cells are often transient cell states that play an important role in biological processes, including cell development and disease progression. Therefore, much effort has been devoted to identifying rare cells. GiniClust (Jiang et al., 2016) is proposed to use Gini index as the criterion for gene selection before performing single cell clustering. It assumed that genes with a high Gini index are highly expressed in a rare cell type. However, due to the nature of Gini index, the selected genes can hardly distinguish between relatively large clusters, which we call abundant cell types. In this paper, we further observe that Gini index calculated globally on the whole data fails in identifying rare cell types that are outlying to local neighbors. Recently, MicroCellClust (Gerniers et al., 2021) is proposed to find rare cell clusters by directly searching the gene-cell subsets with high expression levels in the gene expression matrix, that is, bicluster. This method is effective for the detection of rare cells, but is also not suitable to explore the overall cell population.

Several methods have been developed to solve the problem of detecting rare cells and abundant cell types simultaneously. RaceID (Grün et al., 2015) identifies outlier cells in each cluster after performing k-means to cluster the single cell expression data. It calculates the transcript count probability of each cell, and considers cells whose count probability is below a certain threshold as outlier cells. These outlier cells are then removed from the original clusters and re-clustered. RaceID3 (Herman and Grün, 2018) improves RaceID by adding a feature selection step to reduce the impact of noise on clustering. GiniClust2 (Tsoucas and Yuan, 2018) performs GiniClust to identify rare cell types while uses Fano factor-based k-means (Fano, 1947) to group abundant cell types, and then combine the results through weighted ensemble clustering technique. Further, GiniClust3 (Dong and Yuan, 2020) replaces the clustering algorithm used in GiniClust2 with clustering algorithms with lower time complexity such as Leiden clustering or Louvain clustering. Compared with GiniClust2, it can process large-scale data with higher efficiency. Xie et al. (2020) proposed a deep learning clustering framework called scAIDE. It first uses an Autoencoder to reduce the original data to 256 dimensions, and then performs a random projection hashing based k-means algorithm. However, experiment results show that it still remains a big challenge to detect rare and abundant cell types at the same time.

**FIGURE 1:**
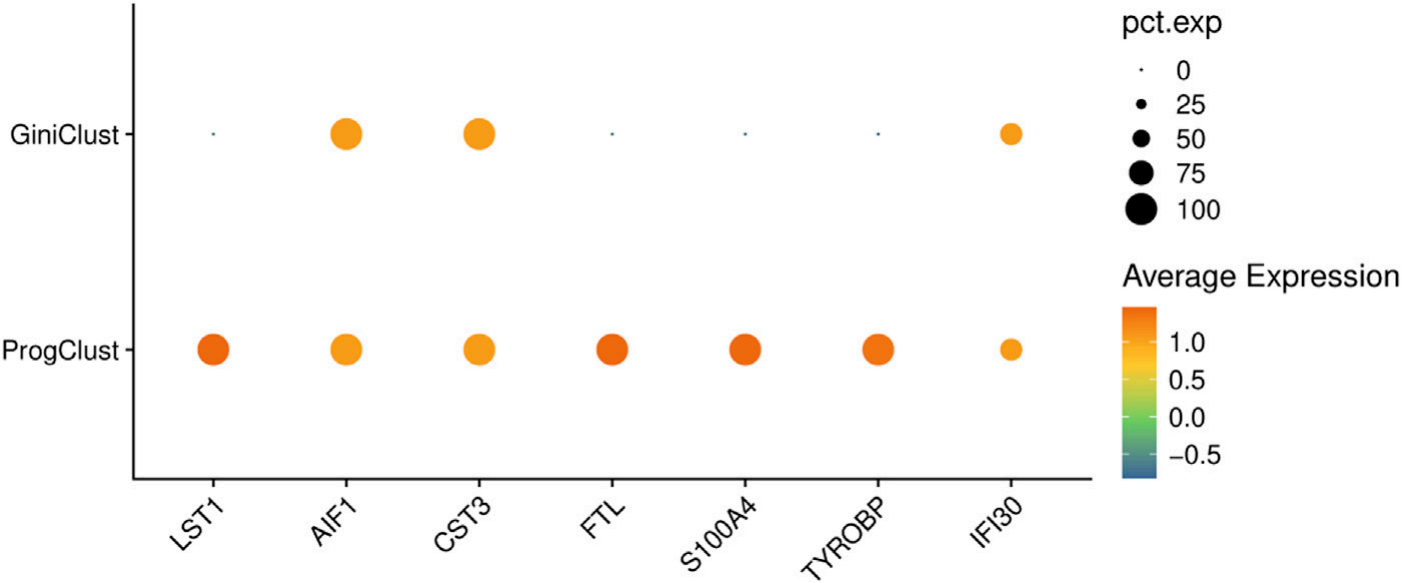
The bubble plots of CD14^+^ Monocyte cell’s marker genes selected by different methods on a sampled data from PBMC68K dataset. ProgClust find 7 gene markers while GiniClust can only find three gene markers.

In order to fully explore the cell populations, in this paper we propose a progressive clustering method, named ProgClust, which can identify the subpopulations of abundant cell types and rare cell types simultaneously. Inspired by previous studies and empirical experiments, we note that gene selection plays a vital role in singe-cell clustering. However, regardless of the feature selection (e.g., gene filter used in SC3, Gini index used in GiniClust) or dimension reduction (e.g., Autoencoder in scAIDE) methods they adopted, previous studies attempts to yield highly expressed genes for abundant cell types or rare cell types on the whole data globally and in one go. Nevertheless, the single-cell data always exhibits complex distributions in cell subpopulations, e.g., local outliers that can not be detected from a global perspective. Consequently, a global gene selection and clustering strategy is not suitable for find clusters with large differences in shape and size. For example, when we try to identify rare cells from a sample that contains five cell types and of which the rare cells, CD14^+^ Monocyte, only accounts for about 0.3% (Zheng et al., 2017), if we use the Gini index to find marker genes for CD14^+^ Monocyte, the genes selected cannot reflect the differences between rare cells and other cells very well. On the contrary, we use ProgClust to progressively explore the subpopluations and use Gini index to find marker genes for CD14^+^ Monocyte, and then obtain more discriminative genes (Figure 1).

ProgClust progressively refines the clusters from coarse to fine by integrating the typical gene selection and clustering methods in a progressive, tree-like pipeline. It starts from the whole cell population as the root node, then performs Fano factor-based gene selection and clustering to group abundant cells into different leaf-nodes which represent cell subpopulations. Subsequently, on each leaf-node, ProgClust performs Fano factor-based clustering as well as Gini index-based rare cell detection locally. When the tree stops growing, all the detected rare cells are then re-clustered in a new tree. Statistical tests are employed to determine the number of clusters and avoid over-clustering. We show that ProgClust can effectively and efficiently identify both abundant and rare cell types of complex single-cell data through extensive experiments.

## 2 Materials and methods

### 2.1 Data preprocessing

In this chapter, there are three publicly available datasets excluding the simulated experimental data. Among them, both PBMC4k and PBMC68k datasets (Zheng et al., 2017) were processed according to the code flow provided by 10X Genomics. Mouse embryonic stem cells were processed according to the process of GiniClust2 (Tsoucas and Yuan, 2018), i.e., filtering out genes expressed on less than three cells and cells expressing less than 2000 genes.

### 2.2 Pipeline of ProgClust

**FIGURE 2:**
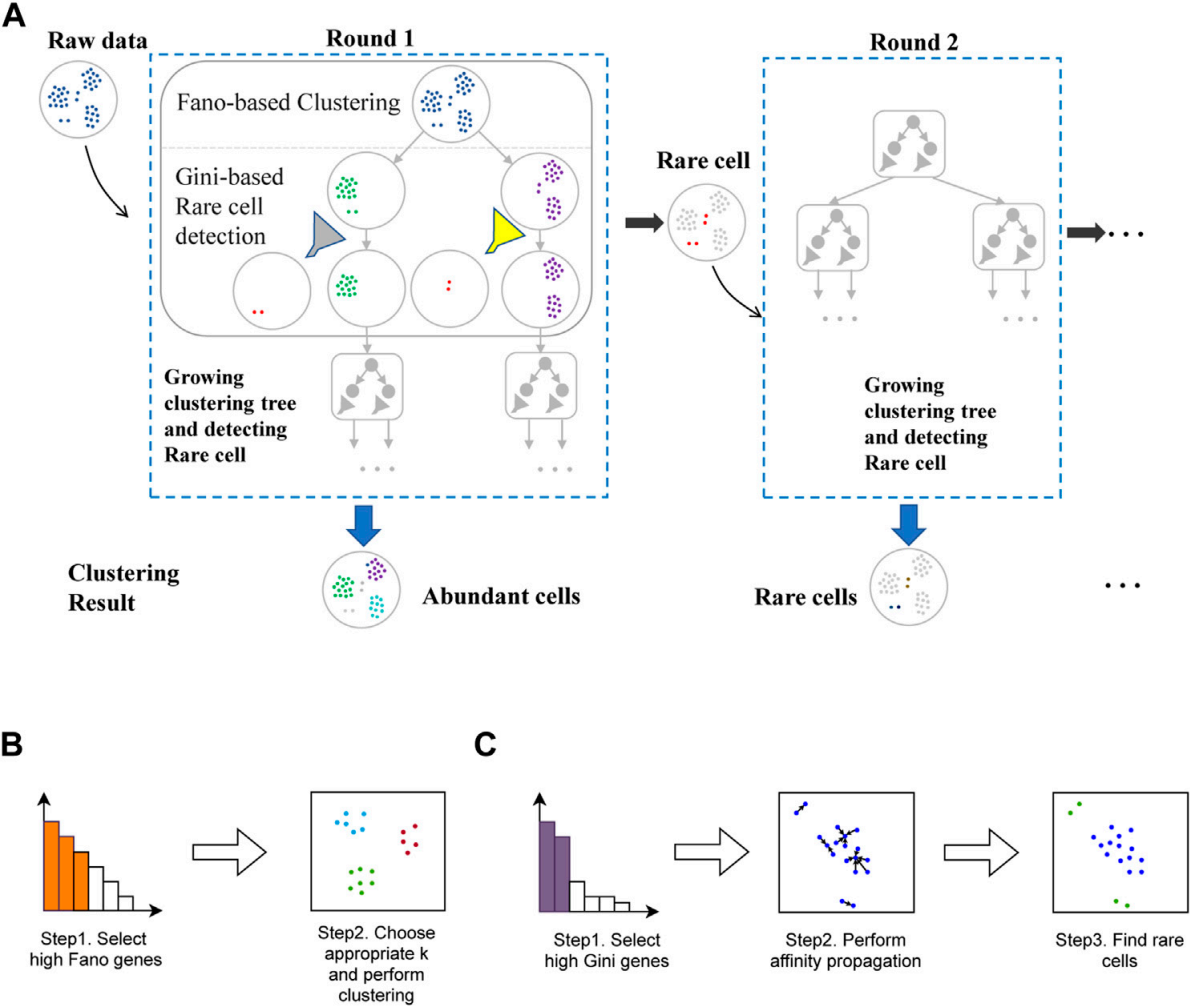
Overview of ProgClust. **(A)** The framework of ProgClust. ScRNA-seq data is input into the first clustering tree. After Fano-based Clustering and Gini-based rare cell detection, the detected rare cells and common cells clustering results are obtained. The rare cells are input to the next clustering tree; **(B)** Fano-based clustering; **(C)** Gini-based Rare cell detection.

Figure 2 displays the pipeline of ProgClust. The methodology is to progressively construct different feature spaces of discriminating genes to distinguish the differences between cells, which finally grows multiple hierarchical clustering trees in different rounds. On each node except the root nodes at the trees, ProgClust performs two steps, Fano-based clustering and Rare cell detection. As shown in Figure 2A, in round 1, at the root node of the first tree, we perform Fano-based clustering, i.e., calculating Fano factors of all the genes using the raw data, then splitting the raw data into several nodes by clustering them based on genes with high Fano factors. In this step, ProgClust identifies the abundant cell types, and splits them to different leaf nodes. Then on each leaf node, we calculates the Gini index of all the genes, and filter out the rare cells mixed in the abundant cells through Gini-based Rare cell detection. By repeating the two steps iteratively (to prevent over-clustering, we iterate twice by default), ProgClust progressively grows a clustering tree of abundant cells and removes rare cells to the next round.

In round 2, the main purpose is to distinguish the rare cell types. ProgClust will first merge all the rare cells into one group, and then construct a new clustering tree following the same pipeline in round 1. Generally, ProgClust can well identify both the abundant cell types and rare cell types in two rounds, i.e., by growing two clustering trees.

### 2.3 Fano-based clustering of abundant cells

First, the Fano factor of each gene will be calculated and the top 1000 genes are selected as features. The Fano factor of each gene is obtained by calculating the ratio of the variance to mean expression value. Let *g*_*i*_ be the expression vector of gene *i* on each cell, var (*g*_*i*_) be the variance of the vector *g*_*i*_, and *mean* (*g*_*i*_) be the mean value of the vector *g*_*i*_, then the Fano factor of gene *i* is defined as:

$$Fano_{i}=\mathrm{var}\left(g_{i}\right)/mean\left(g_{i}\right)$$ (1)

Intuitively, differentially expressed genes are with high Fano factors. Therefore, it can be used in identifying different cell types.

If there is a previous node, the high Fano-factor genes selected in the previous node will be merged into the feature to prevent over-clustering. Then different clustering methods are chosen according to the number of cells. Since the differences between cells are mainly reflected in the differences in expressed genes, the cosine similarity is more able to reflect the differences between cells than the Euclidean distance. But for large samples, k-means based on Euclidean distance is more suitable than spectral clustering based on cosine similarity. When the number of cells is greater than a certain threshold, we will first use PCA (Wold et al., 1987) to reduce the features to 50 dimensions, and then use k-means for clustering. Otherwise, we will first calculate the similarity matrix of the sample based on the cosine similarity, and then perform spectral clustering. We set this threshold to 50.

Since we do not know the number of clusters, we need to determine k according to the structure of the data. For k-means, we use gap statistic (Tibshirani et al., 2001) to determine k, which uses statistical methods to calculate whether there are significant differences before and after clustering. As for spectral clustering, since the similarity matrix has been obtained, it is more convenient to directly determine k through the similarity matrix. Specifically, we judge the choice of k by observing whether the average similarity within the cluster does not increase significantly when k increases. Given a singel cell dataset $X={\left\{x_{i}\right\}}_{1}^{n}$ , let *s* (*i*, *j*) be the similarity between cells, *k* be the number of clusters in the clustering algorithm, and $n\left(C_{m}^{k}\right)$ be the number of cells of the *m*th cluster $C_{m}^{k}$ . Assuming that the *i*th cell belongs to the *m*th cluster when the number of clusters is *k*, the intra-cluster similarity of the cluster $s_{i}^{k}$ where the *i*th cell is located is defined as:

$$s_{i}^{k}=\underset{x_{i}^{\prime }\in C_{m}^{k},x_{j}^{\prime }\in C_{m}^{k},i^{\prime }\neq j^{\prime }}{\sum }s\left(i^{\prime },j^{\prime }\right)/\left(n\left(C_{m}^{k}\right)\left(n\left(C_{m}^{k}\right)-1\right)\right)$$ (2)

We can calculate the $s_{i}^{k}$ of each cell, and define the increase of intra-cluster similarity $r_{m}^{k+1}$ as:

$$r_{m}^{k+1}=\underset{x_{i}\in C_{m}^{k+1}}{\sum }\left(s_{i}^{k+1}-s_{i}^{k}\right)/\underset{x_{i}\in C_{m}^{k+1}}{\sum }s_{i}^{k}$$ (3)

Then, we define the max $r_{m}^{k+1}$ as the similarity gain $S_{k}^{k+1}$ :

$$S_{k}^{k+1}=\underset{m}{\max}\left(r_{m}^{k+1}\right)$$ (4)

We set a gain threshold *ic* (we set it to 0.12 by default, which is used for all my experiments, as are the other parameters.). We start calculating $S_{k}^{k+1}$ from *k* = 1 until $S_{k}^{k+1}$ is less than this threshold, which means that spectral clustering can no longer find new cell types and is unnecessary to increase *k*. The reason for choosing max $r_{m}^{k+1}$ is to encourage the clustering algorithm to be able to find a new cluster, even if the number of cells in this cluster is very small. Sometimes, in order to improve the intra-cluster similarity of certain clusters, the clustering algorithm will reduces the intra-cluster similarity of other clusters. This is obviously detrimental to the clustering result. Therefore, when considering the $S_{k}^{k+1}$ , this part can also be taken into consideration. Assuming that $C_{m^{\prime }}^{k}$ represents those clusters with negative $r_{m}^{k+1}$ , then the similarity gain $S_{k}^{k+1}$ can be defined as:

$$S_{k}^{k+1}=\underset{m}{\max}\left\{r_{m}^{k+1}-\underset{m^{\prime }}{\sum }\left(\frac{n\left(C_{m^{\prime }}^{k+1}\right)}{n\left(C_{m}^{k+1}\right)}r_{m^{\prime }}^{k+1}\right)\right\}$$ (5)

### 2.4 Rare cell detection

Because the variance information of genes differentially expressed on rare cell types can be easily masked by the information on abundant cell types, the fano factor is not suitable for gene selection in rare cell detection. Therefore, it is necessary to detect rare cell in the results of Fano-based clustering.

Given a specific cluster obtained from Fano-based clustering, ProgClust identifies the rare cells and remove them from this cluster with the following three steps.

First, we select locally high Gini genes based on the cells in that cluster, which define the feature subspace for rare cell detection. Let *g*_*ij*_ be the expression value of gene *i* on the *j*th cell, and it has been sorted in ascending sort order, and *n* be the number of cells, then the Gini index can be calculated as:

$$Gini_{i}=\underset{j}{\sum }\left(2j-n-1\right)g_{ij}/\left(n\underset{j}{\sum }g_{ij}\right)$$ (6)

Gini index is originally used to measure the degree of imbalance. Generally, genes with high Gini index are only expressed on a small fraction of cell population, and such genes are particularly useful in distinguishing between rare cell types and abundant cell types.

As illustrated in Figure 1, for a specific cell subpopulation, locally high Gini genes as a whole are more differentially expressed than globally high Gini genes. The local Gini index of each gene is calculated and normalized following the method in (Jiang et al., 2016; Tsoucas and Yuan, 2018). The principal difference is that ProgClust only calculates the raw Gini index on the local data, i.e., the cells in the cluster.

Second, in the defined subspace we calculate the cosine similarities between each cells. We observe that high Gini genes are usually lowly expressed on abundant cells, hence we label the cells whose total expression on the selected genes is below a predefined noise threshold (the default value is 30 for the original count matrix) as abundant cells, which would not be involved in the computation.

Third, based on the similarity matrix, we use Affinity Propagation (Frey and Dueck, 2007) to further partition the subpopulation into multiple smaller clusters. Affinity Propagation is a graph-based clustering algorithm. Compared with other clustering methods such as k-means, Affinity Propagation does not need to preset the number of clusters and is easier to find small clusters in the similarity matrix. Let *s* (*i*, *k*) be the similarity between sample *i* and sample *k*, *r* (*i*, *k*) be the suitability of the sample *k* to become the cluster center of the sample *i*, and *a* (*i*, *k*) be the suitability of the sample *i* to choose the sample *k* to be its cluster center. Then *r* (*i*, *k*) and *a* (*i*, *k*) are calculated as follows:

$$r\left(i,k\right)=s\left(i,k\right)-\underset{k^{\prime }\;s.t\;k^{\prime }\neq k}{\max}\left\{a\left(i,k^{\prime }\right)+s\left(i,k^{\prime }\right)\right\}$$ (7)

$$a\left(i,k\right)=\min\left\{0,r\left(k,k\right)+\underset{i^{\prime }\;s.t\;i^{\prime }\neq \left\{i,k\right\}}{\sum }\max\left\{0,r\left(i^{\prime },k\right)\right\}\right\}$$ (8)

$$a\left(k,k\right)=\underset{i^{\prime }\;s.t\;i^{\prime }\neq k}{\sum }\max\left\{0,r\left(i^{\prime },k\right)\right\}$$ (9)

Affinity Propagation iteratively updates *r* (*i*, *k*) and *a* (*i*, *k*) according to Eq. 7, Eq. 8, Eq. 9 until the algorithm converges, and then identifies clusters according to the result.

To identify rare cell types from these small clusters, we propose a new metric based on the similarity between clusters to evaluate each cluster. Specifically, given the *m*th cluster, let *I*_*m*_ be the average cosine similarity between cells in the cluster and *O*_*m*_ be the average cosine similarity between cells within the *m*th cluster and the cells outside the *m*th cluster, we define the ratio of *I*_*m*_ to *O*_*m*_ as the outlier score of the *m*th cluster. Since rare cell types are generally outlying to the abundant cell types, the outlier score of rare cell clusters should be greater than that of the abundant cell clusters. Here we set a threshold *ros* and clusters with outlier scores greater than *ros* will be considered as rare cell types. Note that the larger *ros* is, the more accurate the rare clusters found, while some rare clusters will be ignored if *ros* is too large. In this paper, we set *ros* = 3.5 by default. In a few cases, there will be a class of clusters with only one cell, and the *I*_*m*_ of that class cannot be calculated. In this case we can only consider *O*_*m*_. We set a threshold *os*, and single-cell clusters with *O*_*m*_ less than *os* will be considered as rare cell types.

## 3 Results

In this section, we show that ProgClust is able to reveal the cell populations without any prior knowledge. We first evaluate the overall clustering performances of ProgClust on two datasets with known labels, in comparison with several state-of-the-art single-cell clustering methods, i.e., GiniClust2, RaceID3, SC3, PARC and scAIDE. The first dataset is a simulation dataset generated by following (Jiang et al., 2016). The second one is the widely used PBMC68k dataset (Zheng et al., 2017). Next, we evaluate the performances of ProgClust in identifying rare cell types. Specifically, we compare the ability of the above methods as well as special clustering algorithms for rare cell detection (i.e., Giniclust, MCC and Gapclust (Fa et al., 2021)) in identifying CD14^+^ Monocyte cells in PBMC68k. Finally, we show how ProgClust effectively detect all cell types on two real unlabeled data, mouse embryonic stem cells (Klein et al., 2015) and PBMC4k dataset.

### 3.1 Evaluation metrics

Considering that the number of clusters may be different from the number of reference cell types, Normalized Mutual Information (NMI) (Strehl and Ghosh, 2002) and Purity are used to measure the clustering performance. NMI is defined as:

$$NMI=\frac{2I\left(X,Y\right)}{H\left(X\right)+H\left(Y\right)}$$ (10)

where *I* (*X*, *Y*) is the mutual information between the clustering result and the reference label, *H*(*X*) and *H*(*Y*) are the information entropy of the clustering result and the reference label, respectively.

Purity is defined as:

$$Purity=\overset{k}{\underset{j=1}{\sum }}\frac{m_{j}}{m}p_{j}$$ (11)

where *k* is the number of clusters, *m*_*j*_ is the number of samples in the *j*th cluster, *m* is the total number of samples, and *p*_*j*_ is the maximum proportion of each type of sample in the *j*th cluster. NMI ranges between 0 and 1, while the range of Purity is [1/*k*, 1], where *k* is the number of reference labels. Note that NMI imposes a small penalty for over-clustering and misclassification, while Purity only pays attention to classification error.

Since we are dealing with unbalanced data, if we assign the same weight to each sample in calculating NMI and Purity, the impact of rare cell types on them will be very small. To fix it, the weight of each sample is the reciprocal of the number of samples in its cluster. When calculating the probability *p*(*C*) of a certain cluster *C*, *p*(*C*) is defined as:

$$P\left(C\right)=\frac{\sum_{x_{i}\in C}w_{i}}{\sum_{i}w_{i}}$$ (12)

where *w*_*i*_ is the weight of the sample *x*_*i*_.

### 3.2 ProgClust accurately clusters simulation data

In order to evaluate the performance of ProgClust, we first generated 4 simulated data following the same method as (Jiang et al., 2016). Number of various types of cells of these four simulated data are shown in Table 1. The first data simulates the situation where a very small number of rare samples are mixed in a large number of samples. The second, third and fourth simulate data simulated a more complex situation. Compared with real data, the boundary between each cluster in the simulated data is clearer. We also evaluated the performance of GiniClust2, SC3, RaceID2, PARC Stassen et al. (2020) and scAIDE, which can automatically select the number of clusters for comparison. In order to show the effect of iteration, we set number of iterations in each clustering tree in ProgClust to 1 and 2, respectively. The former is named ProgClust_deep1, and the latter is directly named ProgClust. The parameters of all algorithms are set according to their recommended parameters.

**TABLE 1:** The number of each type of cells in each simulation data.

Simulation data	Number of cells in each cluster
Simulation data 1	2000,1000,10,6,4,3
Simulation data 2	1000,1000,100,100,10,10
Simulation data 3	1500,1000,1000,100,100,10
Simulation data 4	1500,1000,500,250,100,50

**FIGURE 3:**
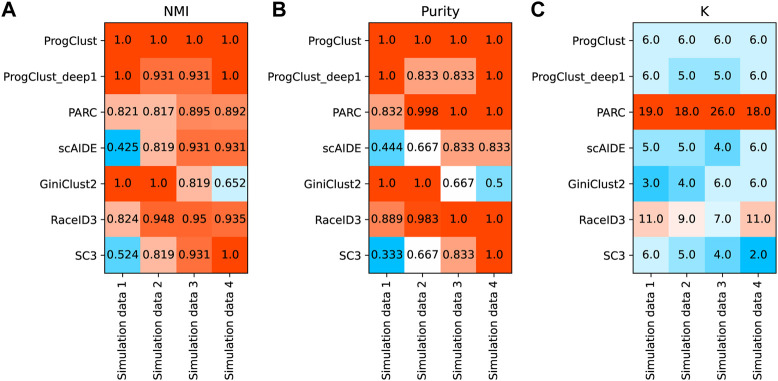
Comparison of the performance of different clustering methods on 4 simulation data. **(A)** NMI of each method on 4 simulation data; **(B)** Purity of each method on 4 simulation data; **(C)** The number of clusters selected by each methods.

Figure 3 reports NMI, Purity and the number of clusters selected by each method on the four simulated data. It can be seen that ProgClust reached the best. Even if the actual value of k is not clear, ProgClust can still correctly find all clusters, while other methods can only accurately classify some clusters. For example, GiniClust2 has a good effect on identifying extremely rare cells, such as Simulated data 1 and Simulated data 2. However, when facing data with more complex distributions, it cannot accurately identify all clusters only by automatically selected k. The other two methods SC3 and scAIDE are the opposite of GiniClust2. They are difficult to identify extremely rare cells, but they can accurately identify all non-rare cell clusters in all datasets. RaceID3 has better robustness in various situations, but it shows over-clustering on these datasets. Therefore, even if the Purity of RaceID3 on both the simulation data 3 and the simulation data 4 reaches 1, the NMI can not reach 1. Similarly, the overall effect of PARC looks good, but it misestimates the number of clusters.

**FIGURE 4:**
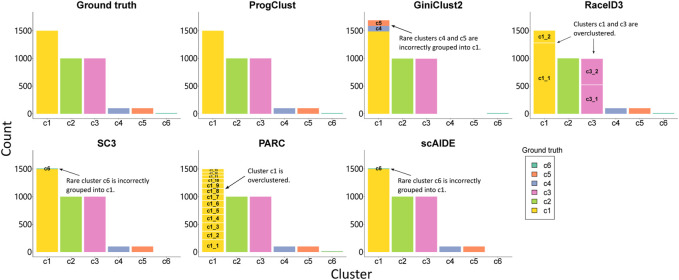
The clustering results of each clustering method on simulation data 3 (other data are provided in the Supplementary Material S1). *X*-axis represents the cluster index, and *y*-axis represents the number of cells. Different colors in the same cluster represent the proportion of different types of cells.

Figure 4 shows the clustering results of each method on the simulation data 3. As expected, ProgClust can accurately identify all clusters. GiniClust2 is able to find the rarest clusters, but it fails to detect the other two rare clusters. Although RaceID3 is able to identify the differences between all cells, including the rarest cells, its over-clustering is obvious and some cells of the same type are divided into two clusters. PARC has the same defect. SC3 detects all common cells perfectly, but it cannot detect rare cells. ScAIDE has the same defect as SC3. These analyses suggest that ProgClust has higher robustness than other methods when the actual k is unknown.

### 3.3 ProgClust is robust in clustering PBMC68k data

In order to evaluate the performance of ProgClust on real data, we design several subsamples by sampling data from PBMC68k dataset (Zheng et al., 2017). PBMC68k dataset contains a total of 65579 cells and can be divided into 11 subpopulations according to transcriptomic similarity with purified cell types. We sample data from 5 subpopulations which are quite different from each other for experiment: CD56^+^ NK cells, CD19^+^ B cells, CD4+/CD25 T Reg cells, CD34^+^ cells and CD14^+^ Monocyte cells. Our sampling process is as follows: First, each cell subpopulation was first further screened based on the expression of the marker genes of that subpopulation to ensure the reliability of the label. The specific screening steps were performed according to the steps given in GiniClust2. (Tsoucas and Yuan, 2018). Then we set a sampling range for each type of cells. After that, the specific sampling number of each type of cells would be randomly selected according to this sampling range. Finally we sampled datasets according to the every cell types’ sampling number. The specific sampling range are shown in Table 2. We sampled 4 types of datasets and repeated sampling 10 times for each type of sampled datasets, and a total of 40 datasets were generated. For each type of sampled data, we take the average performance metric of repeatedly sampled 10 data sets as the result. In this experiment, all algorithms keep the recommended parameters. Since the data has been normalized, we set the noise threshold of our method to 1.

**TABLE 2:** Sampling range of different cell types for each sampled data.

Cell type	CD56^+^ NK	CD19^+^ B	CD4+/CD25 T Reg	CD34^+^	CD14^+^ Monocyte
Sampled data 1	[1000,1929]	[500,1000]	[500,1000]	[10,100]	[3,10]
Sampled data 2	[1000,1929]	[500,1000]	[500,1000]	[3,10]	[3,10]
Sampled data 3	[1000,1929]	[500,1000]	[100,500]	[10,100]	[3,10]
Sampled data 4	[500,1000]	[100,500]	[10,100]	[3,10]	[3,10]

We first evaluated the clustering performance of each algorithm. Table 3 records the results of the experiment. It can be seen that, ProgClust surpasses other algorithms on NMI, while the Purity of RaceID3 is slightly higher than that of ProgClust. However, on all four datasets, RaceID3 selected k which far exceeds the real number of cell types, while our proposed algorithm is more conservative. Obviously, although ProgClust is slightly worse than RaceID3 in Purity, it avoids many meaningless clusters. The performances of GiniClust2, SC3, PARC and scAIDE are all not very good on these four datasets. In addition, we also noticed that the performance of ProgClust_deep1 is not as good as ProgClust, indicating that the iteration of clustering does play a important role. At the same time, we can also find that the gap between the NMI of ProgClust and ProgClust_deep1 is not as large as Purity, which shows that the role of iteration is more reflected in the ability to separate the mixed cells that are not separated in the previous iteration.

**TABLE 3:** Comparison of the performance of different clustering methods on PBMC sampled data: average NMI, Purity and k over 10 independent runs.

Data set	Sampled data 1			Sampled data 2			Sampled data 3			Sampled data 4		
Metrics	NMI	Purity	K	NMI	Purity	k	NMI	Purity	k	NMI	Purity	k
SC3	0.637	0.87	41.6	0.58	0.747	36.9	0.666	0.851	27	0.751	0.805	9.2
RaceID3	0.667	**0.903**	30.2	0.688	**0.92**	33.2	0.677	**0.923**	31.5	0.735	**0.899**	16.2
GiniClust2	0.625	0.639	3.6	0.703	0.689	4.1	0.611	0.682	4.8	0.541	0.875	3.8
scAIDE	0.703	0.727	4.3	0.708	0.708	4.2	0.69	0.724	4.1	0.783	0.812	5.1
PARC	0.731	0.887	10.2	0.702	0.827	9.9	0.719	0.873	9.8	0.75	0.867	8.1
ProgClust_deep1	0.727	0.783	7.5	0.77	0.764	5.5	0.734	0.748	6.1	0.73	0.689	4.5
ProgClust	**0.758**	0.895	11.6	**0.814**	0.888	8.8	**0.8**	0.917	9.8	**0.845**	0.882	6.3

Bold values represent the values with the highest performance metrics on the current data.

### 3.4 ProgClust is robust in identifying CD14^+^ monocyte cells in PBMC68k dataset

Since detecting rare clusters is an important task in single-cell clustering, we also explored the clustering performance of each method on detecting CD14^+^ Monocyte cells, which is the rarest cell type in PBMC68k dataset. Since the number of obtained clusters and the number of real labels are different, there is no metric for evaluating the ability to detect rare clusters and we need to make some adjustments. We divide the clusters that methods identify into two groups, CD14^+^ Monocyte clusters and other clusters. We assume that the clusters with less than 20 cells (twice the upper limit of the number of CD14^+^ Monocyte cells) and containing CD14^+^ Monocyte cells are CD14^+^ Monocyte clusters. We count the number of CD14^+^ Monocyte clusters identified by each method, and then calculate the recall rate and precision rate of CD14^+^ Monocyte cells after merging all CD14^+^ Monocyte clusters. 10 independent runs are performed for each sampled data. Considering that the detection is only for rare cells, we added three algorithms for comparison. The first is MicroCellClust (which is also called MCC), which is a method specifically used to detect rare cells. Since the algorithm can only find one rare cluster at a time, in order to find the cluster to which CD14^+^ Monocyte belongs as much as possible, MCC will run 3 times repeatedly to find 3 rare clusters. The second method is GapClust (Fa et al., 2021). The third algorithm is Gini + ap. This is the part that detects rare cells in ProgClust. Gini + ap first finds rare cells from the original data according to the process of detecting rare clusters in ProgClust, and then performs spectral clustering on these rare cells to find CD14^+^ Monocyte. The purpose of adding this algorithm is to prove that the framework of ProgClust can enhance the detection ability of rare clusters.

Table 4 shows the average performance of different algorithms on identifying rare cells. ProgClust significantly outperforms other algorithms on these data sets, except for RaceID3, and achieve the best Precision and F1 on sampled data 2, sampled data 3 and sampled data 4. Some methods performed poorly, such as SC3 and scAIDE, especially on sampled data 1, sampled data 2 and sampled data 3. On sampled data 4 where rare cells account for a larger proportion, the performence of SC3 and scAIDE will be greatly improved, showing that these methods have limited ability to detect rare cells. The effect of MCC on sampled data 4 is particularly good, but its performance on the first three data is very poor. This may be because MCC can only identify one rare cluster at a time, and this limitation makes it difficult to find the cluster we want in a limited search. The effect of RaceID3 on sampled data 1 is better than ProgClust, which may be caused by over-clustering. In order to prove that our method can achieve better results while allowing over-clustering, we adjusted the parameters of ProgClust (let ros = 2.5). The results after adjusting the parameters are shown in Table 4. It can be seen that the performance of the ProgClust (ros = 2.5) on sampled data 1 and sampled data 2 has been greatly improved and outperforms RaceID3. GiniClust2 and GiniClust performed poorly on these data. These results shows that ProgClust can effectively detect rare cells.

**TABLE 4:** Identification of CD14^+^ Monocyte on 4 sampled data: average value of recall, precision, F1-score and k over 10 independent runs.

Data set	Sampled data 1				Sampled data 2				Sampled data 3				Sampled data 4			
Metrics	Recall	Precision	F1	k	Recall	Precision	F1	k	Recall	Precision	F1	k	Recall	Precision	F1	k
SC3	0.4	0.317	0.351	0.4	0.5	0.324	0.385	0.5	0.181	0.177	0.177	0.3	0.5	0.276	0.345	0.5
GiniClust	0.188	0.3	0.229	0.3	0.526	0.6	0.559	0.6	0.168	0.306	0.208	0.4	0.41	0.6	0.471	0.6
RaceID3	0.92	0.815	0.853	1.1	0.895	0.657	0.714	1.5	**0.96**	0.745	0.799	1.2	0.98	0.763	0.842	1.1
GiniClust2	0	0	0	0	0	0	0	0	0	0	0	0	0.1	0.0778	0.0875	0.2
scAIDE	0.1	0.0571	0.0727	0.1	0	0	0	0	0.229	0.193	0.205	0.3	0.886	0.846	0.862	0.9
PARC	0	0	0	0	0.2	0.141	0.163	0.2	0	0	0	0	0.1	0.0909	0.0952	0.1
GapClust	0.6	0.689	0.689	0.7	**1**	0.935	0.965	1	0.633	0.7	0.655	0.7	**1**	0.979	**0.989**	1
MCC	0.6	0.538	0.567	0.6	0.8	0.702	0.747	0.8	0.5	0.468	0.483	0.5	0.99	0.979	0.984	1
Gini + ap	0.1	0.0909	0.0952	0.1	0.5	0.39	0.432	0.5	0	0	0	0	0.1	0.1	0.1	0.1
ProgClust_deep1	0.6	0.513	0.515	0.7	0.69	0.594	0.616	0.7	0.807	0.58	0.645	1	0.886	0.774	0.813	0.9
ProgClust	0.7	0.775	0.72	0.8	0.978	0.897	0.921	1	0.84	**0.891**	**0.861**	0.9	0.973	**1**	0.986	1
ProgClust [ros = 2.5]	**0.963**	**0.913**	**0.928**	1	0.971	**0.972**	**0.969**	1	0.848	0.737	0.766	0.9	0.962	0.983	0.971	1.1

Bold values represent the values with the highest performance metrics on the current data.

**FIGURE 5:**
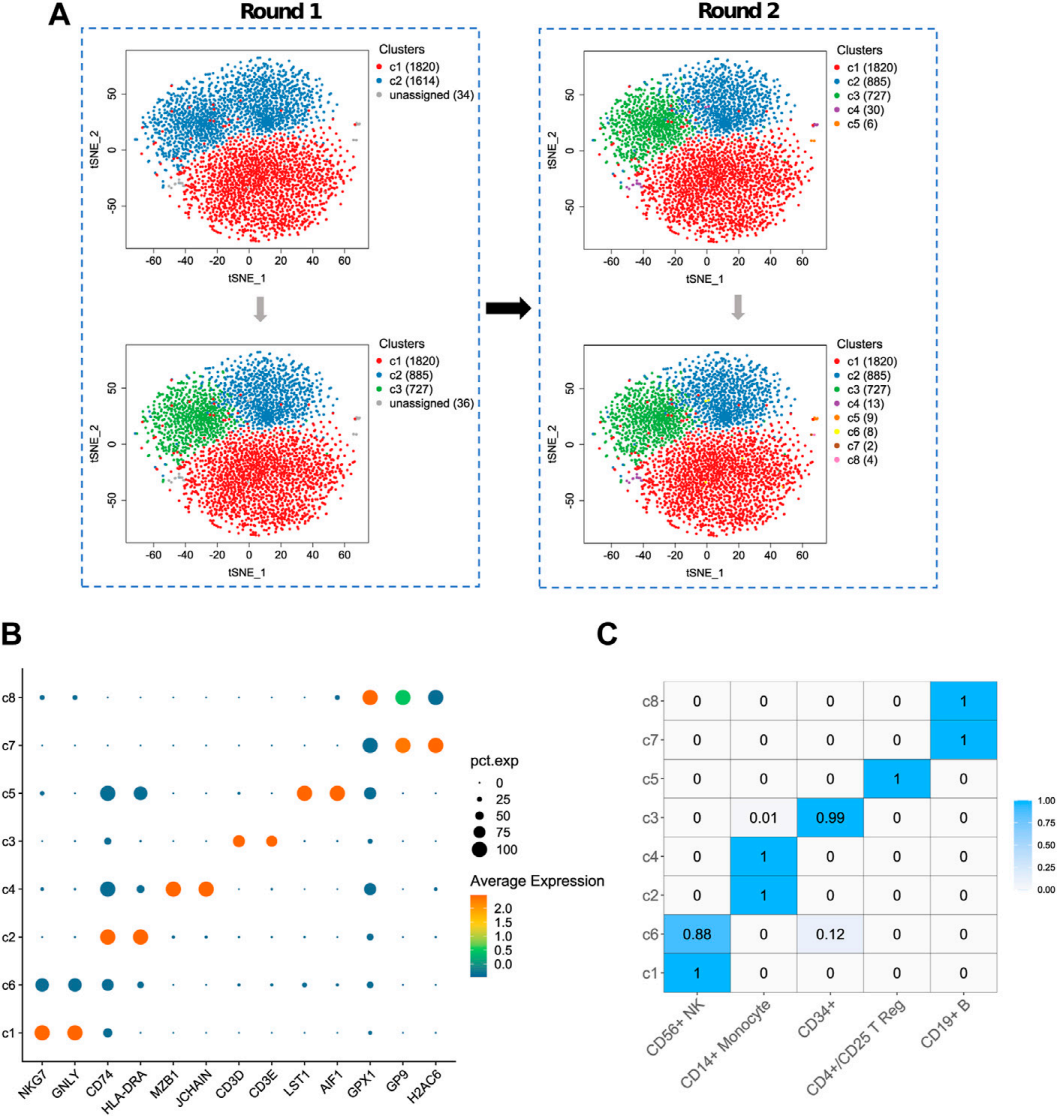
The result of ProgClust on sampled data 2. **(A)** Clustering trees; **(B)** A bubble plot of top differentially expressed genes for each cluster; **(C)** The confusion matrix between the clustering result and the ground truth. The value denotes the proportion of each cell type in the cluster.

Figure 5 shows a result of ProgClust on sampled data 2. In this dataset, according to the reference label, there are 1828 CD56^+^ NK cells, 903 CD19^+^ B cells, 721 CD4+/CD25 T Reg cells, 7 CD34^+^ cells and 9 CD14^+^ Monocytes. The results of each clustering in the ProgClust algorithm process are clearly shown on Figure 5A. ProgClust successfully identified three common clusters in the first clustering tree and found 36 rare cells. And these 36 rare cells were divided into five rare clusters in the second clustering tree. The confusion matrix in Figure 5C shows the percentage of each type of cells in each of the identified class clusters. As can be seen from this matrix, the Purity of the clustering results is still high, with only two class clusters, c3 and c6, containing other cells in addition to the major types of cells. It can also be seen that in this case, over-clustering is also obvious. CD56^+^ NK cells, CD14^+^ Monocyte cells and CD19^+^ B cells are all divided into two cell subpopulations. Differential expression analysis in Figure 5B shows that the identified cell subpopulations are reasonable. For example, although both c2 and c4 belong to CD14^+^ Monocyte cells, c2 clearly has high expression on two genes, CD74 and HLA-DRA, while c4 has two marker genes, MZB1 and JCHAIN, compared to other class clusters, which also implies that ProgClust has identified new cell subpopulations.

### 3.5 ProgClust clusters unlabeled real data

#### 3.5.1 Mouse embryonic stem cells data

We use ProgClust to identify cell types on an unlabeled real data, i.e., mouse embryonic stem cells 4 days after leukemia inhibitory factor withdrawal (Klein et al., 2015). The rare cell types in the dataset have been detected in original publication with the help of priori knowledge of relevant markers, and have been used to test the ability of rare cell detection of singe-cell clustering methods (Tsoucas and Yuan, 2018; Gerniers et al., 2021). In this experiment, we show that ProgClust can reveal the cell subpopulation including the rare cell types without any priori information.

**FIGURE 6:**
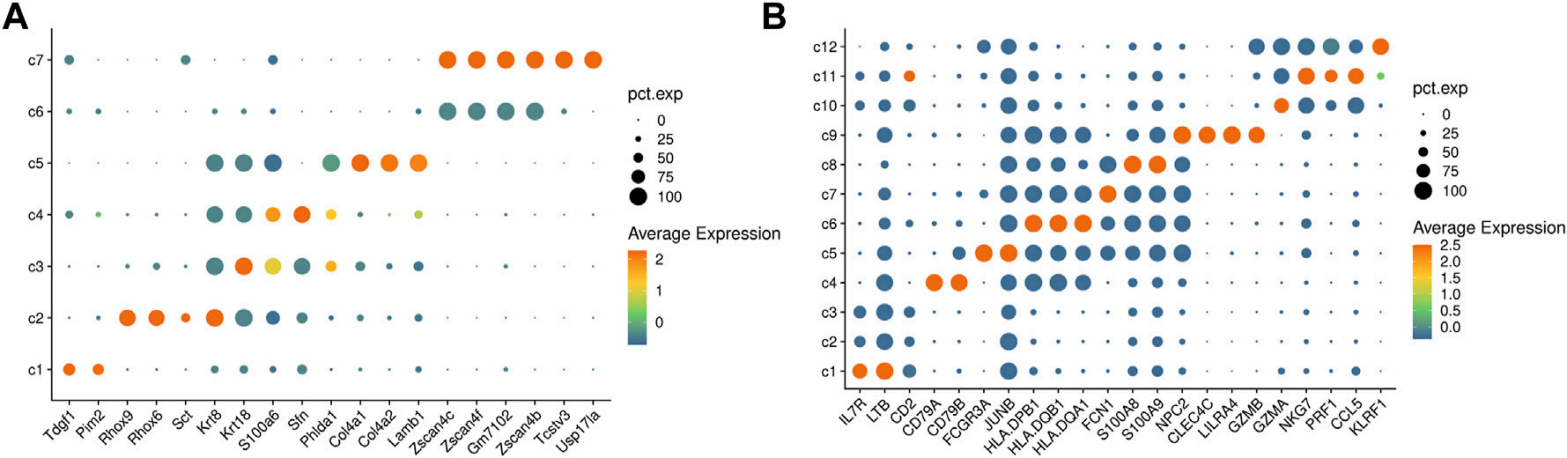
Results from real data analysis. **(A)** A heatmap of differentially expressed genes for the clustering results of the mouse embryonic stem cells data. **(B)** A heatmap of differentially expressed genes for the clustering results of PBMC 4k data.

The dataset contains a total of 683 cells and 24175 genes. ProgClust identifies 4 abundant cell types and 3 rare cell types in the dataset. In Figure 6A, clusters c1 ∼ c4 are abundant cell types and the numbers of them are 349, 34, 101, and 190, respectively. A total of 24 differentially expressed genes in c1 include cell growth and embryonic development related genes such as Pim2 and Tdgf1 (Franzén et al., 2019). The marker genes of c2 are maternal imprinted genes such as Rhox9 and Rhox6. The clusters c3 and c4 have high expression on Krt8, Krt18, and S100a6, indicating that they may be epiblast cells. Clusters c5 ∼ c7 are rare cells, and the number of cells in each cluster are 3, 4, and 2 respectively. Among them, c5 has 12 differential genes, including primitive rare endoderm marker genes such as Col4a1 and Col4a2. The cluster c6 has 27 differentially expressed genes, including marker genes such as Zscan4c and Zscan4f, while c7 has a total of 73 differentially expressed genes, including Zscan4, Zscan4f, Tcstv3 and Usp171a, which indicates that this two clusters correspond to 2C-like cells (Macfarlan et al., 2012). The clustering results of rare cell subpopulation are consistent with previous studies.

#### 3.5.2 PBMC 4K data

We further use ProgClust to explore the cell population of the PBMC 4K dataset from 10x GENOMICS, which contains 4340 cells and 33694 genes. ProgClust identifies 12 clusters in the data. Using differential expression analysis (Figure 6B), we can divide the 12 clusters into 5 cell groups according to the gene markers from PanglaoDB (Franzén et al., 2019). The first one is composed of c1, c2 and c3, with a total of 1804 cells. This group may be T cells, since its marker genes are IL7R, LTB and CD2. The second group is c4, with a total of 623 cells. This group is likely to be B cells, because it strongly expresses genes such as CD79A and CD79B. The third group is composed of c5 ∼ c8. There are a total of 1181 cells in this cell type, and can be characterized by genes related to dendritic cells, such as HLADQB1, FCN1, HLADQA1, etc .,(Villani et al., 2017). The fourth group is c9 and has only 25 cells, which has a strong connection with a number of genes related to plasmacytoid dendritic cells, such as CLEC4C and LILRA4. The fifth group is composed of c10 ∼ c12, which contains a total of 707 cells. The differentially expressed genes are related to NK cells such as NKG7, CCL5 and PRF1. The results show that ProgClust can effectively distinguish these cell types. Furthermore, in the 1st, 3rd and 5th groups, ProgClust also reveals potential cell subpopulations, which provides key clues for further exploration.

## 4 Discussion

The paper presents ProgClust, a novel methodology that utilizes progressive clustering and dynamic feature selection to identify both rare and common cell subpopulations efficiently. Our experiments demonstrate that ProgClust can enhance clustering accuracy effectively, even without prior knowledge of the number of clusters. While our proposed method, ProgClust, has shown promising results, it is not without limitations. Specifically, ProgClust only takes into account cell differences and does not consider cell similarities, which may result in over-clustering and fragmentation of clusters. To address this issue, we plan to investigate two possible solutions in our future research. The first solution involves merging identical clusters *post hoc*, while the second solution involves rejecting clustering results that are more similar than dissimilar during the clustering process. We will focus on developing efficient and effective approaches to implement these solutions to improve the performance of ProgClust.

## Data Availability

The original contributions presented in the study are included in the article/Supplementary Material, further inquiries can be directed to the corresponding author.
