# Supplementary material for: ProgClust: A progressive clustering method to identify cell populations
Source: Front Genet. 2023 Apr 6;14:1183099. doi: 10.3389/fgene.2023.1183099 (PMC10115987; doi:10.3389/fgene.2023.1183099)
Supplement: Supplementary file 2 [file Image1.pdf]

Supplementary Material

1 PARAMETER SENSITIVITY ANALYSIS

The parameters used in our algorithm are mainly three thresholds, ic for determining whether to continue to increase the number of clusters When calculating the number of clusters of Spectral clustering and ros and os for judging whether it is a rare cluster from the result of affinity propagation. We choose datasets from PBMC68k sampled data for experiment. In experiment, except for the test parameters, the other parameters remain default. We used weighted NMI and Purity as evaluation metrics and tested the overall performence and the performence on CD14+ Monocyte cells. Figure S1, Figure S2, Figure S3 and Figure S4 shows the results of this experiment.

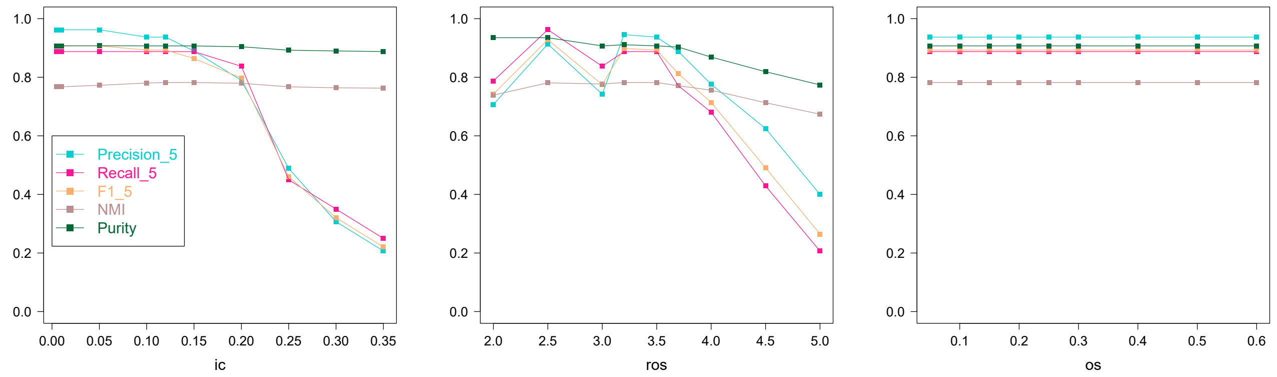

Figure S1. A sensitivity analysis for ProgClust on Sampled data 1. Precesion_5,Recall_5 and F1_5 respectively represent Precesion, Recall and F1-score for performence on CD14+ Monocyte cell, respectively. NMI and Purity represent NMI and Purity for the overall performence, respectively.

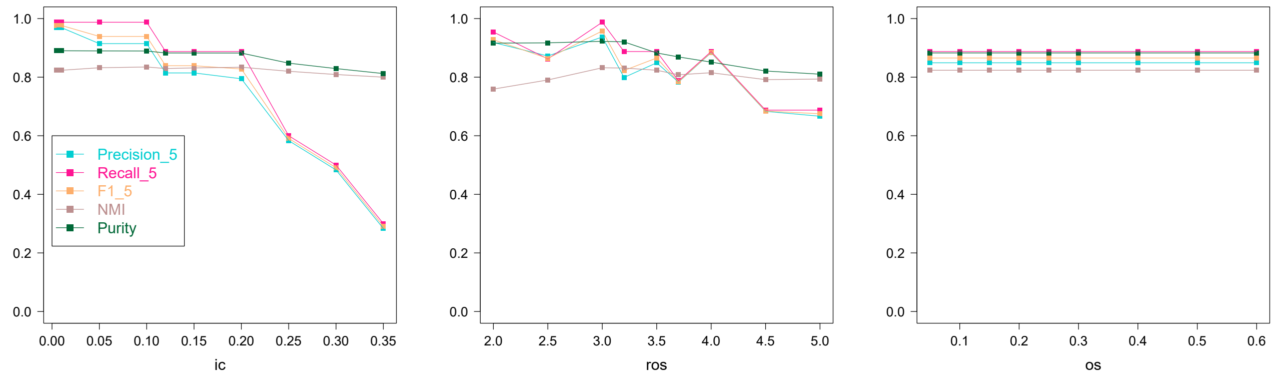

Figure S2. A sensitivity analysis for ProgClust on Sampled data 2. Precesion_5,Recall_5 and F1_5 respectively represent Precesion, Recall and F1-score for performence on CD14+ Monocyte cell, respectively. NMI and Purity represent NMI and Purity for the overall performence, respectively.

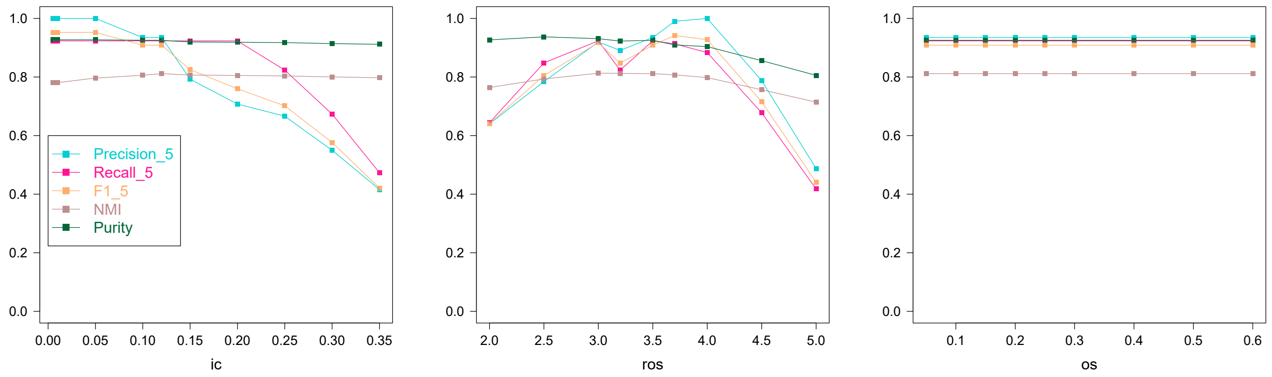

Figure S3. A sensitivity analysis for ProgClust on Sampled data 3. Precesion_5,Recall_5 and F1_5 respectively represent Precesion, Recall and F1-score for performence on CD14+ Monocyte cell, respectively. NMI and Purity represent NMI and Purity for the overall performence, respectively.

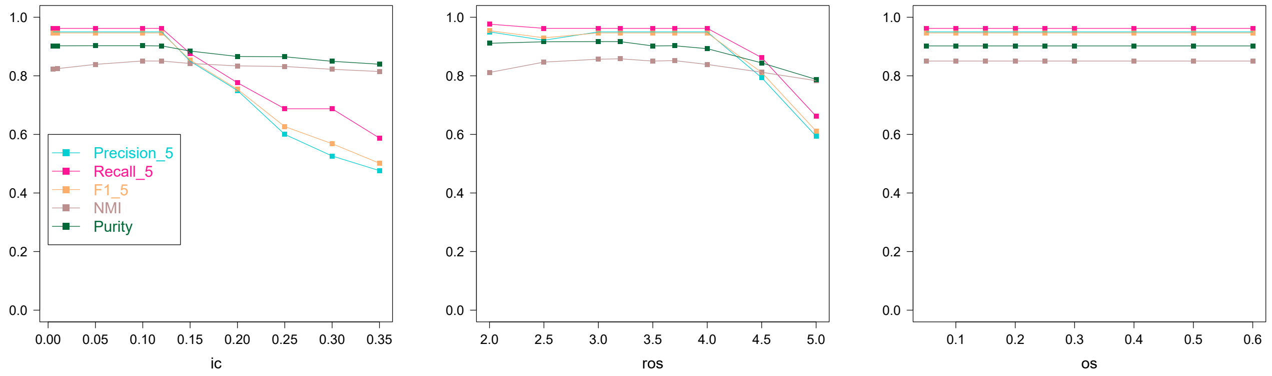

Figure S4. A sensitivity analysis for ProgClust on Sampled data 4. Precesion_5,Recall_5 and F1_5 respectively represent Precesion, Recall and F1-score for performence on CD14+ Monocyte cell, respectively. NMI and Purity represent NMI and Purity for the overall performence, respectively.

It can be seen that os have a small impact on the algorithm performance, while ros and ic has a greater impact. ic has little effect on the detection of whole cells, but has a greater effect on the detection of rare cells. This may be because it only works when dealing with rare cells. Obviously, the smaller the ic, the stronger the ability of ProgClust to detect rare cells. When the ic is less than 0.1, the performance will be more stable. As an important index for detecting rare cells, ros has a great impact on the overall clustering performance and rare cell detection ability. In particular, when ros is between 2.5 and 3.5, ProgClust will have a better performance, and the performance will be greatly reduced above or below this range. In terms of the overall clustering effect, ProgClust has good robustness. Besides, compared to the overall clustering performance, the rare cell cluster detection ability is significantly more sensitive to the parameters, which may be due to the small number of rare cells.

2 ADDITIONAL FIGURES FOR RESULTS ON SIMULATION DATA

Figure S5, Figure S6 and Figure S7 shows the clustering results of each method on the Simulation data 1, Simulation data 2 and Simulation data 4.

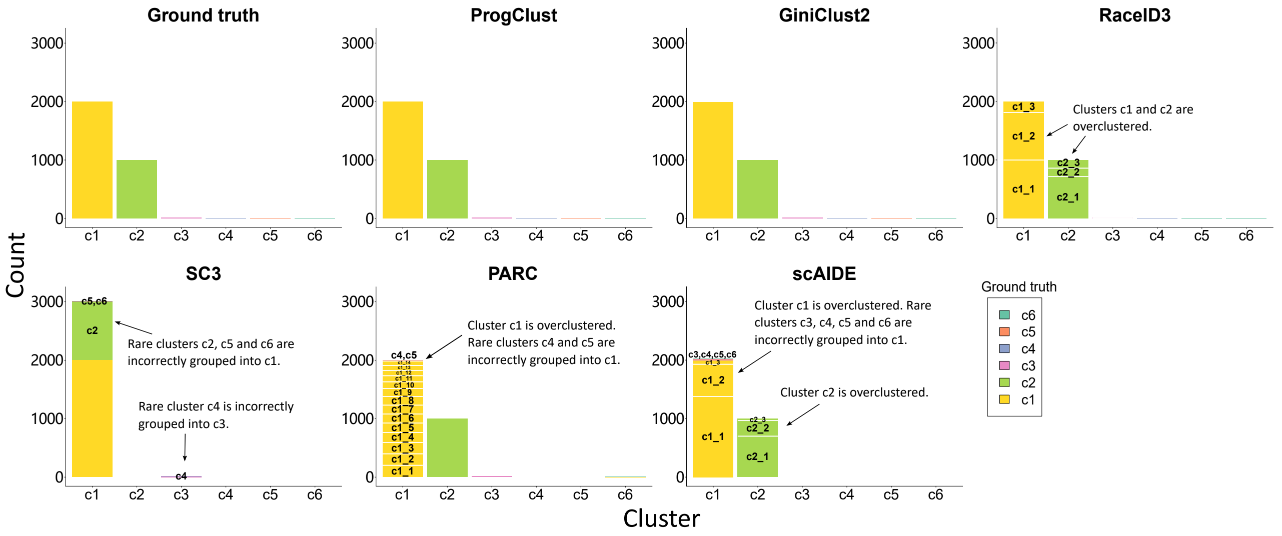

Figure S5. The clustering results of each clustering method on simulation data 1. X-axis represents the cluster index, and y-axis represents the number of cells. Different colors in the same cluster represent the proportion of different types of cells.

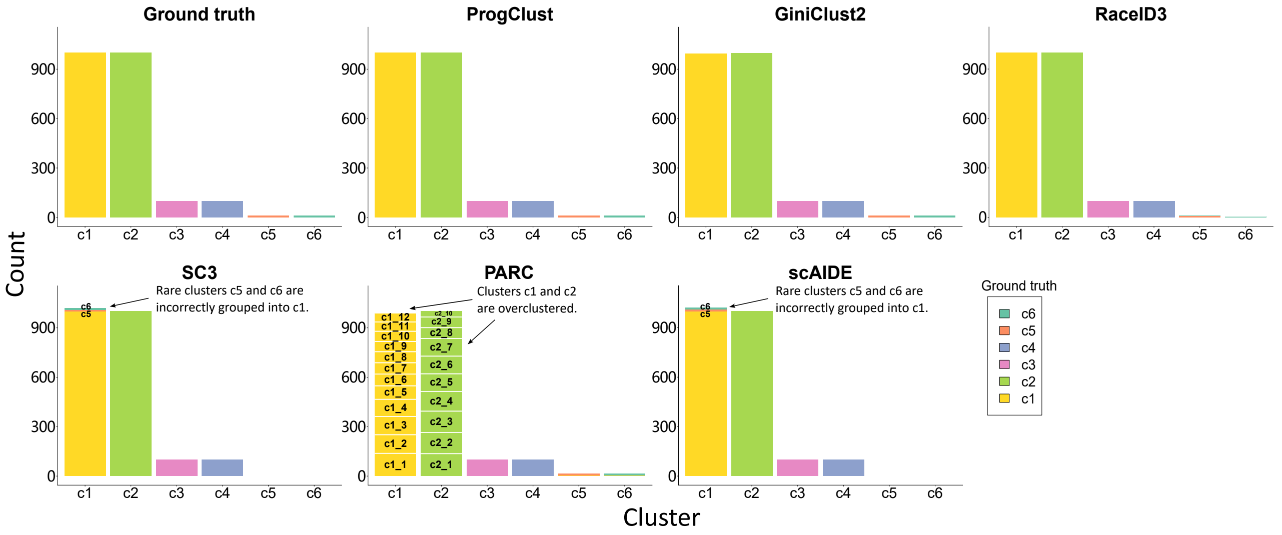

Figure S6. The clustering results of each clustering method on simulation data 2. X-axis represents the cluster index, and y-axis represents the number of cells. Different colors in the same cluster represent the proportion of different types of cells.

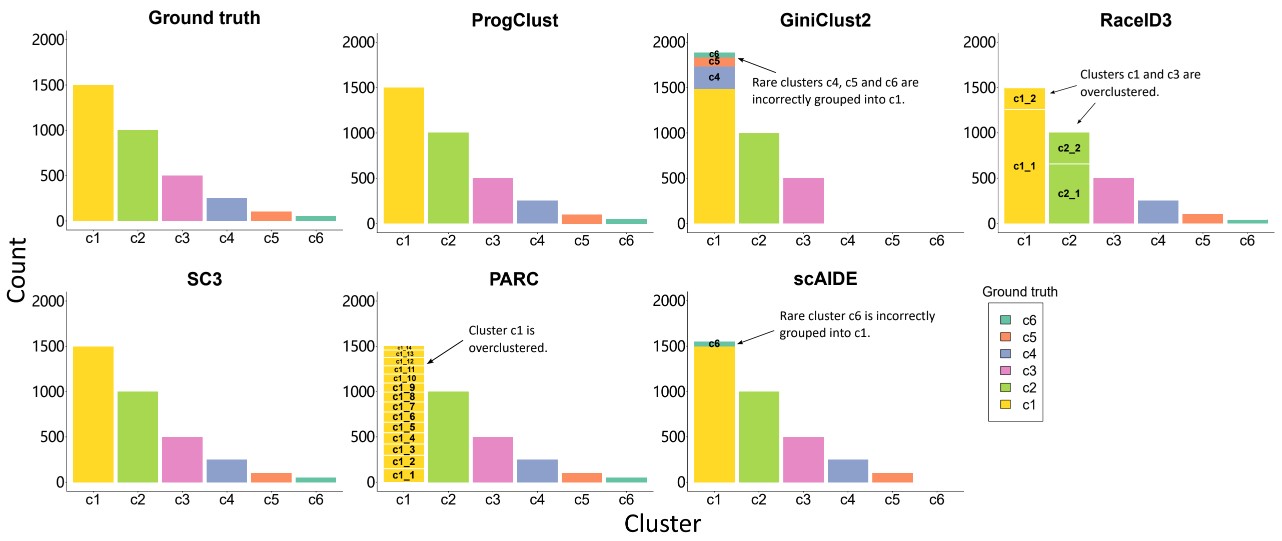

Figure S7. The clustering results of each clustering method on simulation data 4. X-axis represents the cluster index, and y-axis represents the number of cells. Different colors in the same cluster represent the proportion of different types of cells.
